# Early-life hearing loss induces persistent cognitive deficits: evidence from human data and a mouse model with environmental intervention

**DOI:** 10.3389/fnagi.2025.1662732

**Published:** 2025-09-25

**Authors:** Xuehua Zhou, Huiqian Yu, Yiru Wang, Kaizheng Chen, Xia Shen

**Affiliations:** ^1^Department of Anesthesiology, Eye and ENT Hospital, Fudan University, Shanghai, China; ^2^Department of Otorhinolaryngology, ENT Institute, Eye and ENT Hospital, Fudan University, Shanghai, China

**Keywords:** early-life hearing loss, cognitive function, long-term effects, neurodegeneration, enriched environment

## Abstract

Hearing loss during early life has been linked to later cognitive decline, but the underlying neural mechanisms remain unclear. To investigate the cognitive impact of early hearing loss in humans, adults aged 18–40 years with severe childhood hearing loss were evaluated using standardized cognitive assessments. In parallel mouse studies, a mouse model of sensorineural hearing loss was established to assess memory function, hippocampal microglial activation, Tau phosphorylation, and synaptic integrity at 1 and 4 months post-insult. Mice were also exposed to environmental enrichment to test its therapeutic effects. Our data showed that individuals with early hearing loss had a significantly increased risk of cognitive impairment (OR = 1.010, 95% CI: 1.002–1.017, *p* = 0.011). Hearing-impaired mice showed progressive memory deficits, neuroinflammation, Tau hyperphosphorylation, and synaptic loss in the hippocampus. Environmental enrichment improved cognitive performance in affected mice. This study demonstrates that early-life hearing loss induces persistent cognitive deficits in humans, and induces hippocampal pathology in mice. Environmental enrichment effectively improve cognitive performance in affected mice, suggesting that timely interventions may help mitigate adverse outcomes.

## 1 Introduction

Early-life hearing loss (HL) is a common neurodevelopmental condition, affecting approximately 3.1% of children aged 3–19 years (Mehra et al., 2009). In addition to its well-recognized impact on communication, learning, and socialization (Babajanian and Gurgel, 2022), HL during critical developmental windows may exert long-term effects on brain structure and cognitive function. While extensive research has linked age-related HL in adults to cognitive decline and increased dementia risk (Lin, 2011; Lin et al., 2013), the long-term neurocognitive outcomes of HL occurring in early childhood remain insufficiently understood.

Early-life experiences play a pivotal role in shaping brain development and function (Hsiao et al., 2016). Therefore, HL during childhood-a time of heightened neuroplasticity-may disrupt the maturation of key brain regions involved in memory and learning. Neuroimaging studies have reported smaller brain volumes and reduced white matter integrity in individuals with HL (Wang et al., 2022; Croll et al., 2020; Rigters et al., 2017), findings that may underlie slowed cognitive processing (Kuznetsova et al., 2016). Furthermore, HL has been associated with Tau pathology, a hallmark of neurodegenerative disease (Wang et al., 2022).

Animal models further support these findings, demonstrating that noise-or ototoxin-induced cochlear damage impairs hippocampal function and reduces neurogenesis, accompanied by spatial memory deficits (Park et al., 2018). Despite these findings, few studies have examined whether early-life HL leads to sustained cognitive dysfunction and neuropathological changes in the hippocampus. This knowledge gap limits the development of timely interventions to prevent or mitigate cognitive risks associated with early HL.

To address this, we conducted a combined clinical and preclinical study. We first assessed cognitive performance in young adults with a history of severe childhood HL. In parallel, we used a mouse model of early-onset sensorineural HL to investigate hippocampal changes, including microglial activation, Tau phosphorylation, neurogenesis, and synaptic density. We also explored whether environmental enrichment could alleviate cognitive deficits. We hypothesized that early-life HL induces lasting hippocampal pathology and cognitive impairment, which may be phenotypically ameliorated through EE intervention.

## 2 Methods

### 2.1 Clinical investigation

#### 2.1.1 Study design

We conducted a prospective observational study at the Eye & ENT Hospital affiliated with Fudan University, Shanghai, China, between September and December 2024. The study was approved by the Clinical Research Ethics Committee (2024135) and registered in the Chinese Clinical Trial Registry (ChiCTR2400088687, August 23, 2024). Written informed consent was obtained from all participants. The study followed STROBE guidelines.

#### 2.1.2 Enrollment of participants

Patients aged 18 to 40 years were recruited for this study. The cohort included individuals with normal hearing as well as those with hearing loss prior to age 18 who were scheduled for cochlear implantation procedure. Inclusion criteria included the ability to read and speak Chinese, no dementia or severe vision impairment, and a minimum of 6 years of formal education. Data on demographics, medical history, and lifestyle were collected, along with the duration and hearing aid use for those with hearing loss.

#### 2.1.3 Hearing function assessments

Audiometric tests were performed in a soundproof booth. Air conduction thresholds were measured from 0.25 to 8 kHz. A pure-tone average (PTA) was calculated from 0.5 to 4 kHz, and severity was classified per WHO criteria (World Health Organization, 2021).

#### 2.1.4 Cognitive function assessments

Cognitive function was evaluated using the Chinese written versions of the 30-point Montreal Cognitive Assessment (MoCA) (Raymond et al., 2023) and the Digit Symbol Substitution Test (DSST) (Patel and Kurdi, 2015), as previously applied in studies involving patients with hearing loss. Testing began with the MoCA, followed by the DSST for all participants. Personal corrective eyewear was permitted during the assessments, but no additional hearing aids were provided. Testing was conducted in a well-lit, soundproof room to ensure optimal face-to-face communication.

### 2.2 Animal studies

#### 2.2.1 Mice and treatment

The present study was approved by the Animal Care and Use Committee of Fudan University (Shanghai, China) and conducted in compliance with the National Institutes of Health Guide for the Care and Use of Laboratory Animals. Efforts were made to minimize the number of animals used.

Male KunMing mice (postnatal day 7, P7; *n* = 96) were obtained from Vital River Laboratory Animal Technology Co., Ltd. (Beijing, China). To control for potential estrogen effects on cognitive function (Lacreuse, 2006), only male mice were included. The mice were housed in temperature-controlled rooms (22–23 °C) with a 12-h light/dark cycle and free access to food and water. This manuscript adheres to the ARRIVE guidelines for reporting animal research.

#### 2.2.2 Animal model of sensorineural hearing loss

Sensorineural hearing loss model was established using neomycin, as described previously (Yu et al., 2013). Specifically, P7 mice were randomly assigned to either the hearing loss (HL) or control group. The HL group received daily subcutaneous injections of neomycin (200 mg/kg, Sangon Biotech, Shanghai, China) for seven consecutive days, while the control group received an equivalent volume of sterile saline. After treatment, all mice were returned to their mothers until weaning (P28). Behavioral tests were performed at P30 and P120 (*n* = 12 per group). Auditory function was assessed using the auditory brainstem response (ABR) test at P30 and P120 as previously described (Yu et al., 2013). To measure the hearing threshold, ABR analysis was performed on mice following anaesthetisation with an intraperitoneal injection of ketamine (100 mg/kg) and xylazine sodium (25 mg/kg).

#### 2.2.3 Cognitive function assessments

The passive avoidance test and the Y-maze were employed to evaluate cognitive function (*n* = 12 per group) (Kim et al., 2020; Kwon et al., 2018). The passive avoidance test apparatus consisted of light and dark compartments separated by an automated door. The test included two phases: training and testing. During training, each mouse was placed in the light compartment, and after a 30-s adaptation period, the door opened automatically. The latency to enter the dark compartment was recorded. Upon entry, the door closed, and a brief electrical shock (0.7 mA, 2 s) was administered. In the test phase (24 h later), mice underwent the same procedure without the shock, and the latency to enter the dark compartment was recorded (maximum 300 s). The spontaneous alternation behavior of mice was evaluated using a standard Y-maze setup. Each mouse was placed in the center of the Y-maze and allowed to explore freely for 8 min. The number of consecutive entries into different arms (alternations) was recorded, and the percentage of spontaneous alternations was calculated as an indicator of working memory.

#### 2.2.4 Brain tissue harvest

Mice were anesthetized with esketamine (50 mg/kg, i.p.) and xylazine (25 mg/kg, i.p) and perfused intracardially with cold phosphate-buffered saline (PBS) for 10–15 min, followed by 4% paraformaldehyde (20–30 mL). The brains were post-fixed overnight at 4 °C and dehydrated in 30% sucrose. Hippocampal sections (20–40 μm thick) were prepared using a freezing microtome (Leica, Wetzlar, Germany) and stored in a cryoprotectant solution (30% sucrose and 30% ethylene glycol in 0.1 M PBS) at −20 °C. Three sections of the dentate gyrus (DG), spaced 320 μm apart, were selected per animal for analysis of neurogenesis and microglial activation.

#### 2.2.5 Immunohistochemistry

Free-floating brain sections were selected for Doublecortin (DCX) and Iba1 staining (*n* = 3 per group). After 30 min of PBS wash, sections were blocked with 10% goat serum and 0.5% Triton X-100 in PBS for 2 h, followed by overnight incubation at 4 °C with primary antibodies: anti-DCX (1:2000; Abcam, ab18723) and anti-Iba1 (1:200; Cell Signaling, #17198S). After PBS washing, sections were incubated with Alexa-647 goat anti-rabbit secondary antibody (1:2000; Abcam, ab150083) for 2 h, then stained with DAPI (1:1000; Sigma, D9542) in the dark. DCX images were captured using a confocal microscope (Leica, 40 × objective, 2 μm z-step, 1,024 × 1,024 resolution), and Iba1 images were obtained with a whole-slide scanner (3DHISTECH). Cell counting was performed using ImageJ software. The number of DCX-positive cells in selected areas was quantified to assess neurogenesis, and the percentage of Iba1-stained area in the dentate gyrus was measured for microglial activation.

#### 2.2.6 Western blot analysis

Mice were sacrificed at postnatal day 30 (P30) or postnatal day 120 (P120) (*n* = 6 per group). Bilateral hippocampi were quickly extracted and lysed using pre-cooled tissue lysates with protease inhibitors. Total protein concentrations in the supernatant were determined using a bicinchoninic acid (BCA) assay (Beyotime Biotechnology, Shanghai, China). Equal amounts of protein were separated on 10% or 15% sodium dodecyl sulfate-polyacrylamide gel electrophoresis (SDS-PAGE) gels and transferred onto polyvinylidene difluoride (PVDF) membranes. After blocking with a quick blocking reagent (Beyotime Biotechnology) for 15 min, the membranes were incubated overnight at 4 °C with the following primary antibodies: Snap25 (1:10000; Synaptic Systems, Göttingen, Germany; 111,002); Synaptophysin 1 (SYN1, 1:10000; Synaptic Systems; 101,002); Tau5 (1:500; Invitrogen, Waltham, MA, United States; AHB0042); Phosphorylated tau (P-Tau, Ser396, 1:1000; Abcam, Cambridge, United Kingdom; ab109390); PSD95 (1:1000; Cell Signaling Technology, Beverly, MA, USA; #3450S); NR2B (1:1000; Cell Signaling Technology; #4212S); IL-1β (1:1000; Cell Signaling Technology; #12242S); TNF-*α* (1:1000; Cell Signaling Technology; #11948); GAPDH (1:5000; Proteintech, Wuhan, China; HRP-60004); and Tubulin (1:5000; Proteintech; HRP-66031). After washing with TBST (Tris-buffered saline with 0.1% Tween-20), the membranes were incubated with horseradish peroxidase (HRP)-conjugated secondary antibodies (goat anti-mouse IgG-HRP and goat anti-rabbit IgG-HRP; West Grove, PA, USA) at a 1:10000 dilution for 1.5 h at room temperature. GAPDH or Tubulin was used as a loading control to normalize protein levels. Mice were sacrificed at P30 or P120. Bilateral hippocampi were quickly extracted and lysed with tissue lysis buffer containing protease inhibitors. Protein concentrations were determined using a BCA assay (Beyotime). Equal protein amounts were separated by 10–15% SDS-PAGE and transferred to PVDF membranes. After blocking for 15 min, membranes were incubated overnight at 4 °C with primary antibodies: Snap25 (1:10,000), SYN1 (1:10,000), Tau5 (1:500), P-Tau Ser396 (1:1000), PSD95 (1:1000), NR2B (1:1000), IL-1*β* (1:1000), TNF-*α* (1:1000), GAPDH (1:5000), and Tubulin (1:5000). Membranes were washed and incubated with HRP-conjugated secondary antibodies (1:10,000) for 1.5 h at room temperature. GAPDH or Tubulin was used as a loading control.

#### 2.2.7 qRT-PCR

Total RNA was extracted from hippocampal tissues using TRIzol (Thermo Fisher) (*n* = 3 per group). cDNA was synthesized with the PrimeScript™ RT Kit (TaKaRa). Quantitative PCR was performed using TB Green® Premix Ex Taq™ (TaKaRa) on an ABI 7500 system. Relative gene expression was calculated using the 2^−ΔΔCT method with *β*-actin as the normalization control. Primer sequences: IL-1β (Forward: 5′-TACATCAGCACCTCACAAGC-3′, Reverse: 5′-AGAAACAGTCCAGCCCATACT-3′); TNF-α (Forward: 5′-TCGTAGCAAACCACCAAGTG-3′, Reverse: 5′-AGATAGCAAATCGGCTGACG-3′); β-actin (Forward: 5′-CCTCTATGCCAACACAGT-3′, Reverse: 5′-AGCCACCAATCCACACAG-3′).

#### 2.2.8 Enriched environment

The enriched environment (EE) procedure in this study was adapted from previous protocols [18] with specific modifications. Mice were housed in a 70×70×46 cm cage with 5–6 toys (wheels, ladders, mazes). From P12 to P120, mice were exposed to the EE for 2 h daily. Toys were rearranged 2–3 times weekly to maintain novelty (n = 10 per group). Supplementary File 1 provides comprehensive environmental enrichment (EE) protocol details, including several images of representative items.

#### 2.2.9 Statistical analysis

Previous studies found that the mean MoCA scores for individuals with HL and those with normal hearing were 24.3 (2.33) and 26 (2.56) (Shen et al., 2013), respectively. With a significance level of 0.05 and a power of 0.90, at least 36 subjects per group are needed to detect significant differences. To assess the relationship between HL, cognitive impairment, and factors like age, BMI, education, and duration of HL, we used multiple regression analyses. Given the inclusion of five predictors, we aimed for a larger sample size to ensure reliable results and sufficient statistical power. Therefore, we planned to include 100 subjects in each group to support the study’s objectives.

Normality of continuous variables was assessed with the Shapiro–Wilk test. Normally distributed variables are presented as mean ± SEM and compared using Student’s t-test. Non-normally distributed continuous variables are presented as median and interquartile ranges and compared using the Mann–Whitney *U* test. Categorical variables are presented as counts and percentages of the total and were compared using the Chi-square test or Fisher’s exact test, as appropriate. Multiple logistic regression analyses were performed to assess associations, adjusting for age, gender, education level, duration of HL, hearing function, and hearing aid usage. Odds ratios (ORs) with 95% confidence intervals (CIs) were reported. For behavioral studies, 10–12 mice per group were used, and for biochemical studies, 3–6 mice per group, based on prior research (Dupuis et al., 2015). Differences between control and HL groups were determined using a two-tailed unpaired Student’s *t*-test or the Mann–Whitney *U* test. A *p* value <0.05 was considered significant. Statistical analyses were conducted using GraphPad Prism (version 8.0) and SPSS (version 25).

## 3 Results

### 3.1 Hearing function was associated with cognitive impairment in patients

A total of 224 participants were screened for eligibility. Of these, 13 individuals with hearing loss and 11 with normal hearing function declined to complete the cognitive screening. Consequently, 200 participants were successfully enrolled (Supplementary Figure 1). The median MoCA score of the participants with hearing loss was significantly lower than that of the participants with normal hearing function (23.5 vs. 26.2, *p* < 0.001). Similarly, the mean DSST scores of the participants with hearing loss was lower than that of the participants with normal hearing (43.7 vs. 51.2, *p* < 0.001). Multiple logistic regression analysis revealed that hearing loss [OR (95%CI): 1.010 (1.002–1.017), *p*=0.011], age [OR (95%CI): 1.071 (1.025–1.118), *p*=0.002] and low educational level (educational year less than 9 years) [OR (95%CI): 10.721 (3.037–37.843), *p*<0.001] were the risk factors for the development of cognitive impairment (Table 1).

**Table 1:** Association between hearing loss and cognitive impairment.

Factors	OR (95% CI)	*p*-value
BMI	1.039 (0.948; 1.139)	0.408
Age (yr)	1.071 (1.025; 1.118)	0.002
Duration of hearing loss (yr)	1.001 (0.950; 1.053)	0.983
Educational year <9 years	10.721 (3.037; 37.843)	<0.001
Hearing loss (decibels)	1.010 (1.002; 1.017)	0.011
Severe hearing loss (PTA≥50)	0.213 (0.009; 4.945)	0.335

BMI, Body mass index; PTA, Pure tone average; OR, Odd ratio.

### 3.2 Hearing loss induces cognitive impairment in mice

**Figure 1:**
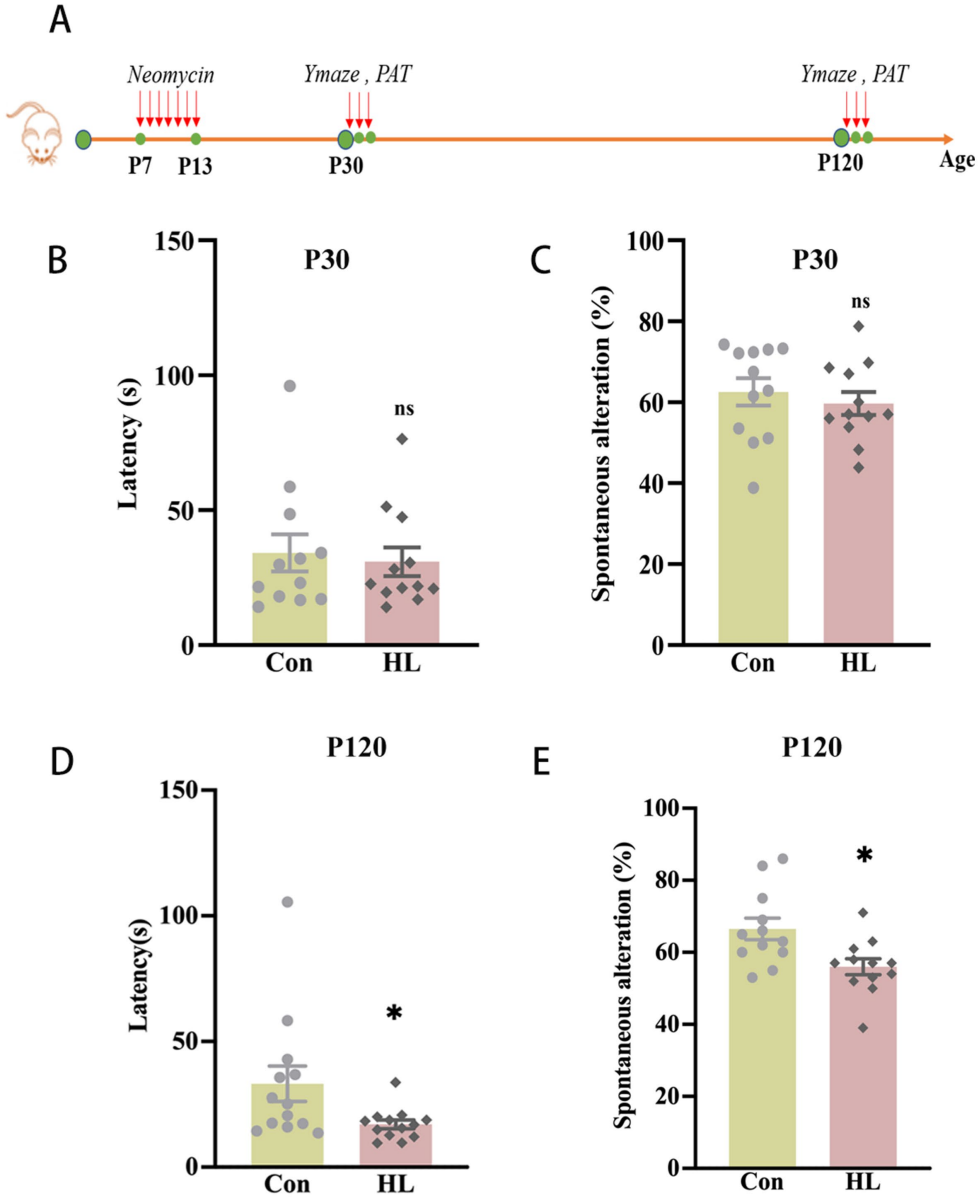
Cognitive dysfunction occurs later in adult mice with HL. The experimental design for the passive avoidance test and Y maze test **(A)**. At P30, there was no significant difference in the latency of entering the dark room in PAT **(B)** or the percentage of correct alternations out of the total number of alternations in the Y maze test between the Control and HL groups **(C)**. At P120, the latency of the HL group was significantly shorter than that of the Control group in PAT **(D)**. The percentage of correct alternations out of the total number of alternations in the HL group was significantly lower than that of the Control group in the Y maze test **(E)**. Data are shown as the mean ± SEM. ns, *p >* 0.05; *p < 0.05. Con, control; HL, hearing loss.

HL in mice was induced by neomycin. Auditory brainstem response (ABR) thresholds were significantly higher in the HL group than in controls at postnatal days 30 (P30) and 120 (P120), with a greater increase at P120 (Supplementary Figure 2). The passive avoidance test (PAT) and the Y maze test were conducted at P30 and P120 to evaluate cognitive function in mice (Figure 1A). At P30, there was no significant difference between groups in latency to enter the dark compartment in the PAT (30.92 ± 5.01 s vs. 34.18 ± 6.85 s, *p* = 0.711; Figure 1B) or alternations in the Y maze (59.95% ± 4.80 vs. 62.55% ± 3.38, *p* = 0.657; Figure 1C). However, at P120, the mice with HL exhibited significantly shorter latencies in PAT (33.17 ± 7.05 s vs. 17.04 ± 1.735 s, *p* = 0.025; Figure 1D) and significantly lower percentage of correct alternations (56.00% ± 2.24 vs. 65.38% ± 2.99, *p* = 0.021; Figure 1E) in the Y maze. Together, these findings from the PAT and Y maze test suggest that early-life HL impairs working memory later in life in the mice.

### 3.3 Hearing loss induces neuroinflammation and microglial activation in hippocampus of mice

**Figure 2:**
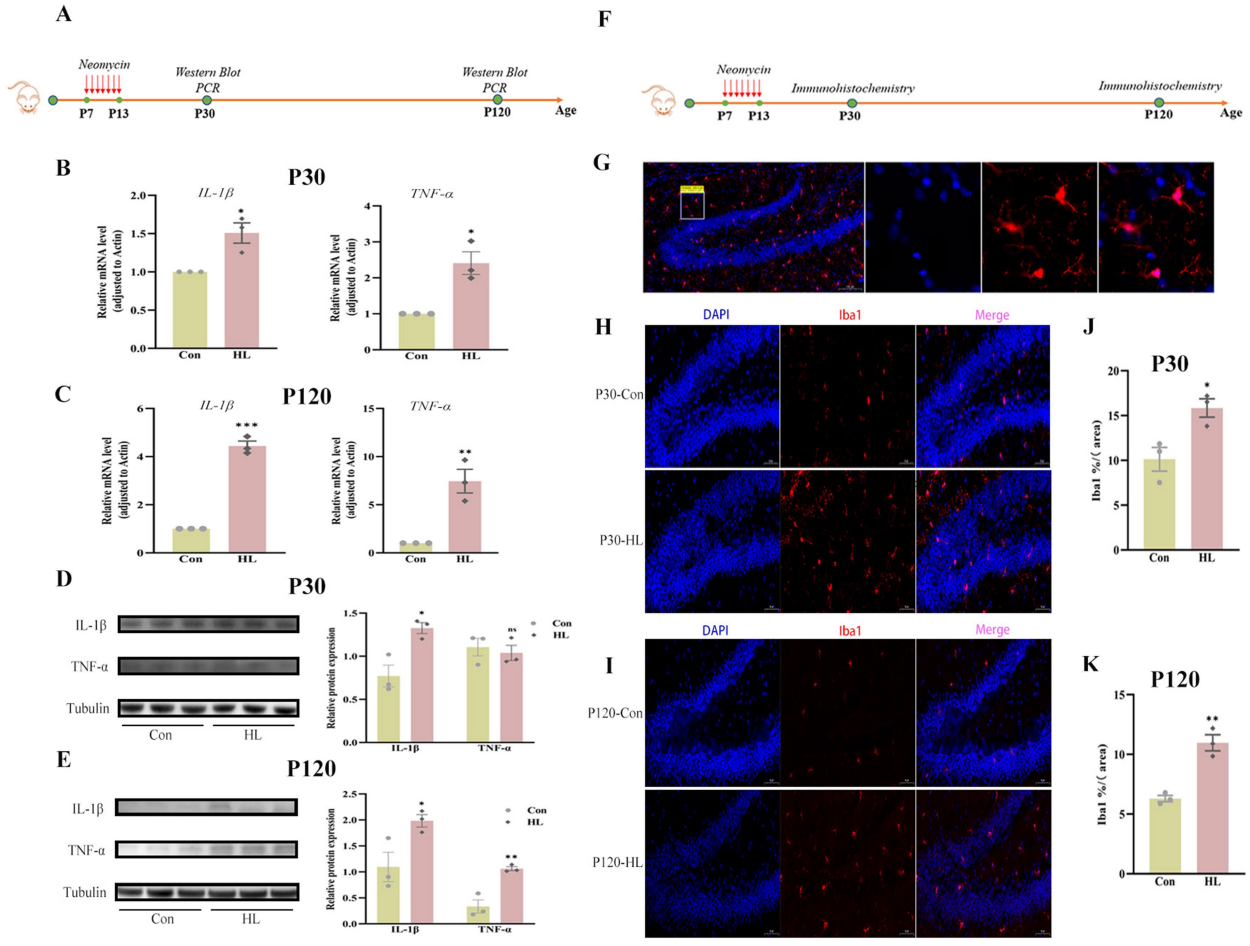
Increased neuroinflammation and microglial activation occur in the hippocampus in HL mice. The experimental design for detecting the expression of inflammatory factors using western blot and qRT-PCR **(A)**. Mice in the HL group have increased expression of IL-1β and TNF-*α* in the hippocampus at P30 **(B)** and P120 **(C)**. At P30, the western blot image and comparison date showed that the expression of IL-1β increased in HL mice, whereas the TNF-α level was not significantly different between the two groups **(D)**. At P120, both the western blot image and qRT-PCR analysis showed increased expression levels of IL-1β and TNF-α in the hippocampus in HL mice **(E)**. The experimental design for observing the activation of microglia using immunohistochemistry **(F)**. Representative images of DAPI (blue) and Iba1 (red) immunostaining in the hippocampal dentate gyrus (DG) region. The right panel shows magnified views of the boxed areas, highlighting microglial presence. Scale bar, 50 μm **(G)**. Quantification of Iba1 expression demonstrated increased microglial activation in the HL group at both P30 **(H)** and P120 **(I)**. The percentage of Iba1-positive staining area was significantly higher in HL mice compared to controls at both P30 **(J)** and P120 **(K)**, indicating persistent microglial activation. Data are presented as mean ± SEM. ns, *p* > 0.05; *p < 0.05; **p < 0.01; ***p < 0.001. Con, control; HL, hearing loss; DAPI, 4′,6-diamidino-2-phenylindole; Iba1, ionized calcium-binding adapter molecule 1; DG, dentate gyrus.

Research has highlighted the deleterious effects of neuroinflammation in cognitive function (Li et al., 2023; Rump and Adamzik, 2022). In this study, we examined the expressions of interleukin-1β (IL-1β) and tumor necrosis factor-*α* (TNF-α) in the hippocampus of mice (Figure 2A). qRT-PCR study revealed significant increases in IL-1β (1.51 ± 0.13 vs. 1.0, *p* = 0.019) and TNF-α (2.41 ± 0.31 vs. 1.0, *p* = 0.046) in the HL group compared to the controls at P30 (Figure 2B). At P120, these levels remained elevated (IL-1β: 4.44 ± 0.21 vs. 1.0, *p* = 0.004; TNF-α: 7.45 ± 1.23 vs. 1.0, *p* = 0.006; Figure 2C). Western blotting confirmed the increases in IL-1β at both P30 (1.32 ± 0.06 vs. 0.77 ± 0.13, *p* = 0.017) and P120 (1.98 ± 0.12 vs. 1.09 ± 0.28, *p* = 0.044; Figures 2D,E). There were no significant differences in the TNF-α concentrations at P30 (1.04 ± 0.09 vs. 1.11 ± 0.10, *p* = 0.643; Figure 2D). However, P120 mice with HL had significantly higher levels (1.06 ± 0.04 vs. 0.34 ± 0.13, *p* = 0.005; Figure 2E).

Neuroinflammation includes microglia activation (Singh, 2022; Isik et al., 2023). We examined the expressions of the activation of microglia in the hippocampus at P30 and P120 (Figure 2F). Upon activation, microglia exhibit larger soma and shorter, thicker branches, a morphology associated with neurodegenerative diseases. In the present study, microglial activation, assessed by Iba1 staining (Figure 2G), showed larger activated areas in the dentate gyrus at both P30 (15.85 ± 1.03 vs. 10.12 ± 1.33, *p* = 0.027) (Figures 2H,J) and P120 (10.97 ± 0.68 vs. 6.29 ± 0.26, *p* = 0.003; Figures 2I,K) in HL mice compared to controls. These results show that HL triggers neuroinflammation in the hippocampus, which progresses over time, e.g., P30 versus P120.

### 3.4 Hearing loss induces tau phosphorylation in hippocampus of mice

**Figure 3:**
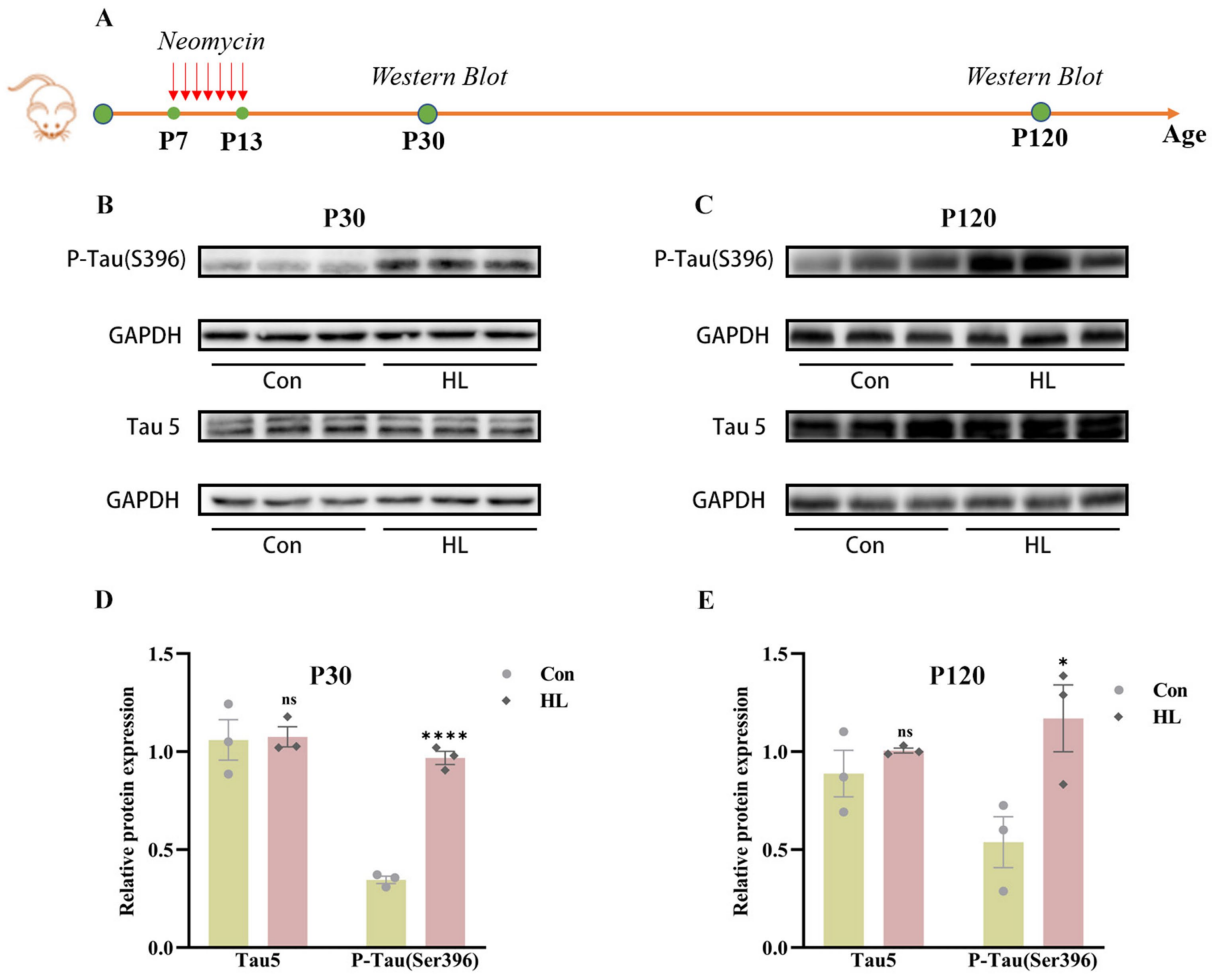
Tau protein accumulates in the hippocampus of mice after HL. The experimental design for detecting the expression of Tau protein using western blotting **(A)**. Western blotting results showed that the expression of P-Tau (S396) in the hippocampus was increased in HL mice, whereas Tau5 (total Tau) was not different between the two groups at P30 **(B)** and P120 **(C)**. Quantification of western blots for P-Tau (S396) and Tau5 at P30 **(D)** and P120 **(E)**. Data are shown as the mean ± SEM. ns, *p >*0.05; *p < 0.05; ****p < 0.0001. Con, control; HL, hearing loss.

Tau is essential for microtubule stability and its abnormal aggregation is linked to neurodegenerative diseases like Alzheimer’s (Digma et al., 2019). To investigate how HL induces cognitive impairment, we measured total Tau (Tau5) and phosphorylated Tau (P-Tau, Ser396) concentration, which is related to cognitive decline (Liu et al., 2022), in the hippocampus of mice at P30 and P120 (Figure 3A). As early as P30, P-Tau was significantly elevated in mice with HL (0.97 ± 0.03 vs. 0.35± 0.02, *p* <0.0001), while Tau5 levels were comparable (1.08 ± 0.05 vs. 1.06 ± 0.10, *p* = 0.900; Figures 3B,D). This elevation in P-Tau concentration persisted at P120 (1.17 ± 0.17 vs. 0.54 ± 0.13, *p* = 0.042), with Tau5 levels remaining unchanged (1.01 ± 0.01 vs. 0.89 ± 0.12, *p* = 0.379; Figures 3C,E). These findings suggest that the accumulation of P-Tau may contribute to the HL-induced cognitive impairment in mice.

### 3.5 Hearing loss reduces neurogenesis and synaptic integrity in hippocampus of mice

**Figure 4:**
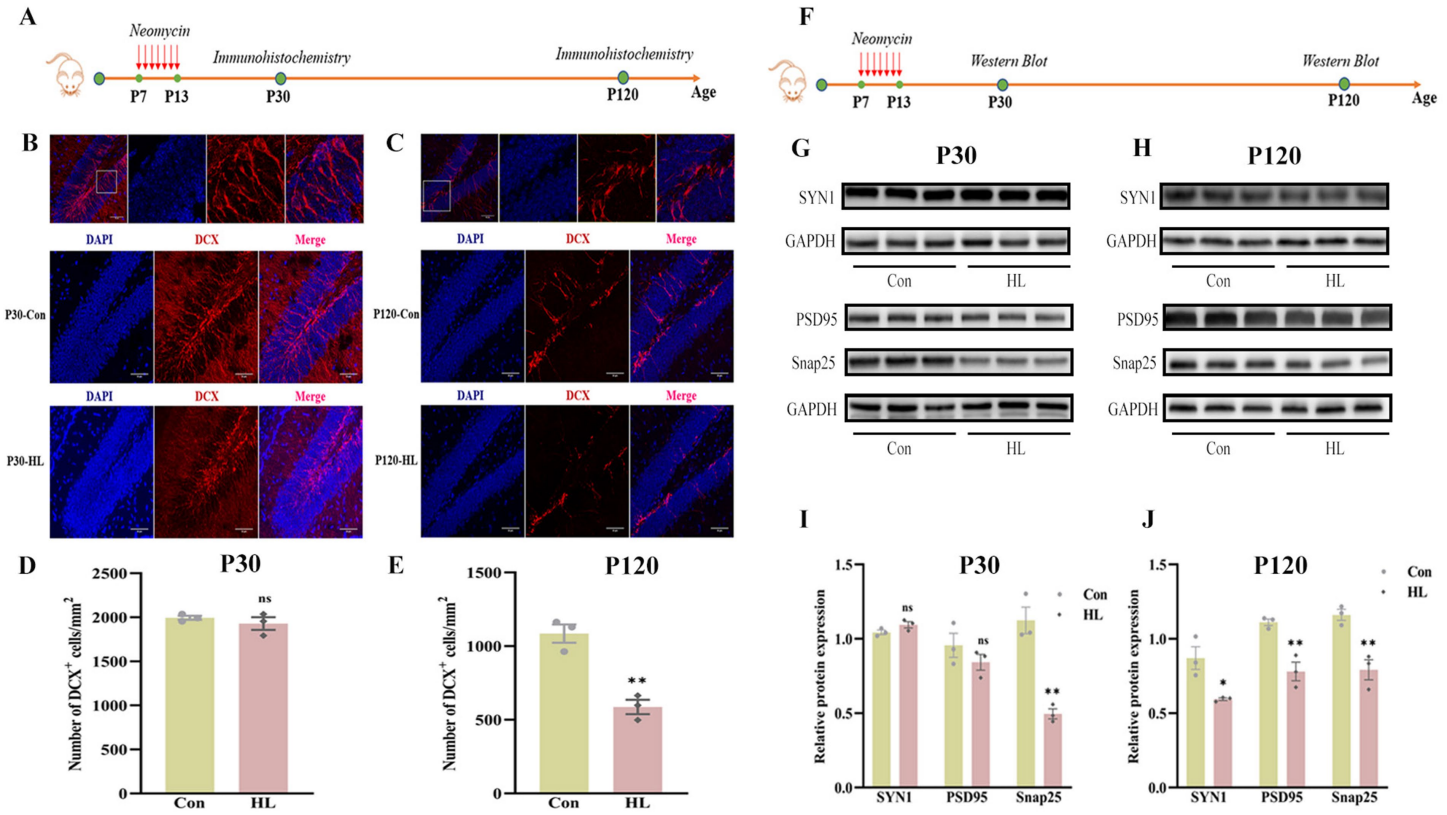
Neurogenesis and synaptic integrity decline in the hippocampus in adult HL mice. The experimental design for evaluating hippocampal neurogenesis using immunohistochemistry **(A)**. At P30, there were no significant differences in the density and morphology of DCX+ cells in the hippocampus between the two groups. The upper image indicates the representative images of the enlarged DG area in the white box. DAPI (blue); DCX (red). Scale bar, 50 μm **(B)**. At P120, HL mice exhibited a significant reduction in DCX+ cells compared to controls, with shorter dendrites that failed to integrate into the outer layer. Representative images of the DG region are shown in the upper panel. Scale bar, 50 μm **(C)**. Quantification of DCX+ cell density at P30 **(D)** and P120 **(E)**, revealing a significant decline in neurogenesis over time in HL mice. Experimental design for detecting synapse-associated proteins using western blot **(F)**. At P30, the expression of Snap25 was significantly reduced in HL mice, while Synaptophysin (SYN1) and PSD95 levels remained unchanged compared to controls **(G)**. At P120, HL mice exhibited a significant reduction in the expression levels of Snap25, Synaptophysin, and PSD95 **(H)**. Quantification of western blot results showing expression levels of Snap25, SYN1, and PSD95 at P30 **(I)** and P120 **(J)**, indicating progressive synaptic impairment in HL mice. Data are presented as mean ± SEM and analyzed using an unpaired t-test. ns, *p* > 0.05; *p < 0.05; **p < 0.01. Con, control; HL, hearing loss; DAPI, 4′,6-diamidino-2-phenylindole; DCX, doublecortin; DG, dentate gyrus; Snap25, synaptosomal-associated protein 25 kDa; SYN1, synaptophysin 1; PSD95, post-synaptic density protein 95.

Reduced neurogenesis in the hippocampus is associated with cognitive impairment (Geigenmuller et al., 2024; Li et al., 2021). In the present study, we assessed neurogenesis by detecting doublecortin (DCX), a marker of immature neurons in hippocampus of mice (Figure 4A). At P30, no significant differences were observed in the number or dendritic morphology of DCX+ neurons between HL and control groups (1928.19 ± 71.85 vs. 1994.85.0 ± 23.55, *p* = 0.428; Figures 4B,D). Neurogenesis naturally declines with age, a trend confirmed in our experiment, as shown by immunofluorescence results (Figures 4B,D). By P120, however, the mice with HL displayed significantly fewer DCX+ neurons and shorter dendritic branches than controls (586.82 ± 48.26.59 vs. 1,085 ± 61.64, *p* =0.003; Figures 4C,E), suggesting a decline in neurogenesis over time.

We also analyzed synaptic markers (Snap25, SYN I, and PSD95), which are linked to cognitive decline (Wang et al., 2023; Liu et al., 2020; Qi et al., 2024; Figure 4F). At P30, Snap25 was significantly lower in the HL group (0.50 ± 0.03 vs. 1.12 ± 0.09, *p* = 0.003) (Figures 4G,I). By P120, Snap25 (0.79 ± 0.07 vs. 1.16 ± 0.04, *p* = 0.009), SYN I (0.59 ± 0.01 vs. 0.87 ± 0.08, *p* = 0.023), and PSD95 (0.78 ± 0.06 vs. 1.11 ± 0.02, *p* = 0.007) were all significantly decreased in the HL group (Figures 4H,J). These findings suggest that HL may cause potential synaptic loss or dysfunction, as reflected by decreased expression of synaptic markers (PSD95, Synapsin-1), contributing to cognitive impairment.

### 3.6 Environmental enrichment mitigates hearing loss-induced cognitive impairment in mice

**Figure 5:**
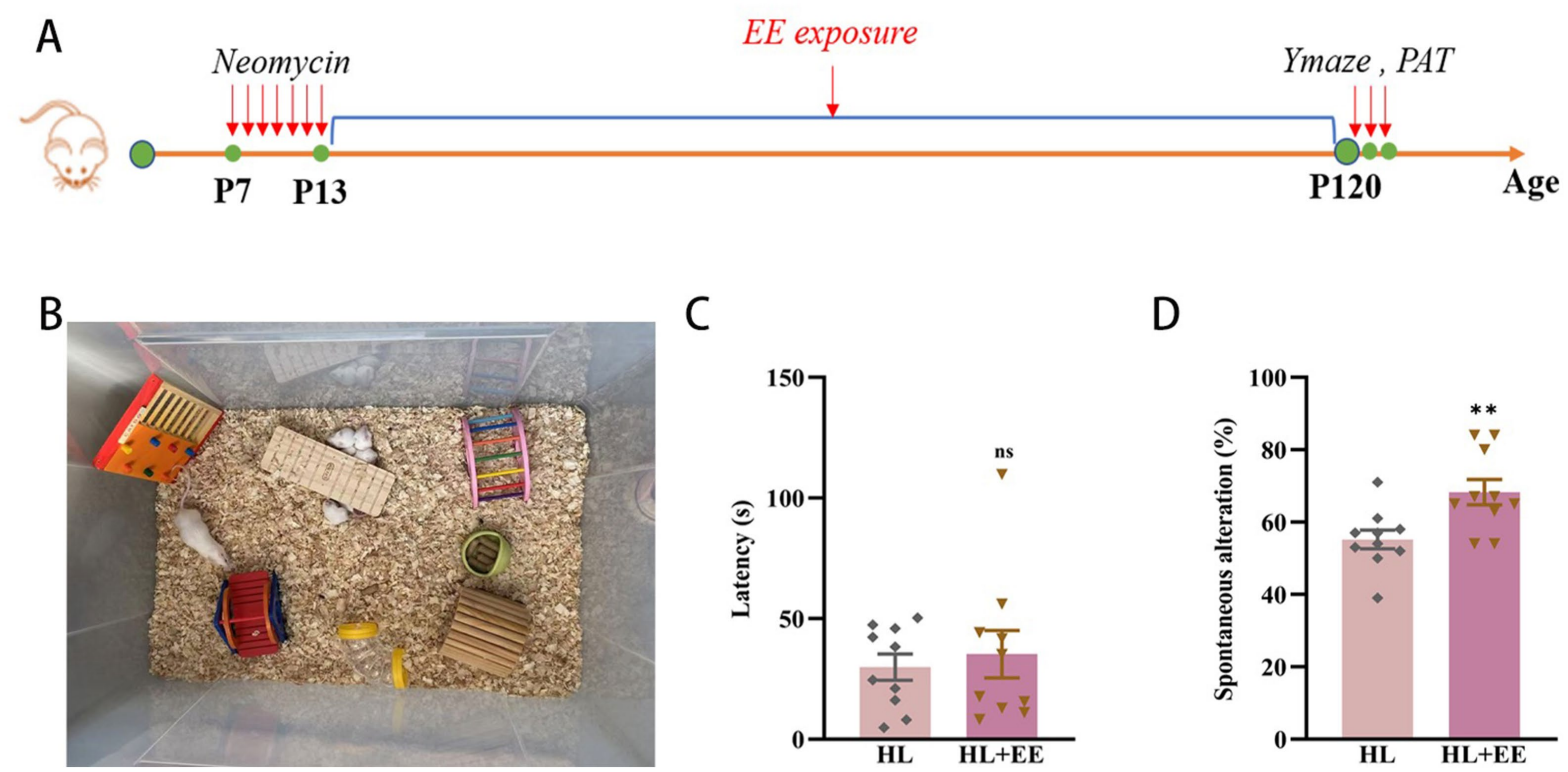
Environmental enrichment attenuates cognitive impairment in hearing loss mice. The experimental design for environment enrichment **(A)**. Illustration of environment enrichment **(B)**. The latency of the HL+EE group was similar to that of the HL group in PAT test **(C)**. The percentage of correct alternations out of the total number of alternations in the HL group was significantly lower than that of the HL+EE group in the Y maze test **(D)**. Data are shown as the mean ± SEM. ns, *p >* 0.05; **p < 0.001. Con, control; HL, hearing loss; EE, environmental enrichment; PAT, passive avoidance test.

This clinical study demonstrated that higher education appeared to protect against cognitive decline in patients with HL. Similarly, prior research has shown that environmental enrichment (EE) enhances cognitive function in animal models (Shen et al., 2013). To investigate whether EE could mitigate HL-induced cognitive impairment in mice, we conducted assessments at P120 (Figures 5A,B). No significant difference was observed in latency to enter the dark compartment in the PAT (35.26 ± 9.79 s vs. 28.95 ± 5.93 s, *p* = 0.599) (Figure 5C). However, correct alternations in the Y maze were significantly improved in the EE group (68.30% ± 3.48 vs. 55.20% ± 2.59, *p* = 0.007) (Figure 5D). These findings suggest that EE can partially ameliorate hippocampal-dependent short-term memory impairment associated with HL in mice, rather than long-term memory deficits. This distinction may reflect differential sensitivity of memory circuits to enrichment, and further studies with larger cohorts are warranted to confirm this observation.

## 4 Discussion

### 4.1 Early-life hearing loss and cognitive impairment: insights from clinical and animal studies

This proof-of-concept study establishes a significant association between early-life hearing loss and cognitive impairment in adulthood, as demonstrated by findings in patients and in mice. Key pathological changes observed in the hippocampus of mice with hearing loss include Tau protein phosphorylation, reduced neurogenesis, impaired synapses, and heightened neuroinflammation, suggesting these mechanisms contribute to the hearing loss-induced cognitive impairment.

Sensory deprivation during critical developmental periods, particularly before sexual maturity, can cause permanent disruptions in brain function (De Villers-Sidani et al., 2007). Sensory stimulation is crucial for modifying neural connectivity and strengthening cognitive abilities during brain development (Chaudhury et al., 2013). Studies have demonstrated a relationship between hearing and cognitive function in children (Ronnberg et al., 2014), with temporary conductive hearing loss in early life leading to persistent cognitive and memory deficits (Williams and Jacobs, 2009). Similarly, neonatal mice exposed to intense noise experience severe hearing loss, chronic spatial learning and memory deficits, and reduced neurogenesis months after the exposure (Tao et al., 2015). These findings from the previous studies are consistent with the results from the present study that hearing loss in early life can lead to cognitive impairment in later times.

Multiple analysis in the present study revealed that hearing loss and older age were associated with lower MoCA scores, consistent with the results from a previous study showing that prolonged hearing loss is a predictor and risk factor of cognitive impairment (Lin et al., 2013). These findings emphasize the long-term impact of untreated hearing loss on cognitive decline. This discrepancy may stem from the high proportion of participants with severe to profound hearing loss in the present study. Additionally, higher educational attainment was found to protect against cognitive impairment, supporting the cognitive reserve hypothesis, which posits that education enhances resilience to brain pathology (Lovden et al., 2020).

### 4.2 Mechanistic insights from the mouse model

The data from the animal studies provided valuable insights into the mechanisms underlying cognitive impairment associated with hearing loss. Early hippocampal neurodegenerative changes were evident by 1 month, characterized by increased P-Tau (S396) accumulation, reduced Snap25 expression, and elevated neuroinflammation. These changes preceded detectable cognitive impairment, indicating that hippocampal pathology begins before the appearance of cognitive impairment. By 4 months, these changes became more pronounced, with further reductions in neuronal markers such as doublecortin-positive cells, dendritic branches, and synaptic proteins (SYN1 and PSD95).

Tau protein phosphorylation may disrupt neuronal signaling (Weingarten et al., 1975). Reduced neurogenesis impairs the generation of new neurons, affecting learning and memory function.^27^ A decline in synaptic proteins reflects diminished synaptic connections, impairing information processing efficiency (Fuentes-Santamaria et al., 2007). Elevated neuroinflammation exacerbates neuronal damage, collectively contributing to cognitive impairment (Leng and Edison, 2021). These interacting mechanisms highlight the complex pathology of hearing loss-induced cognitive impairment, pending confirmation studies in the future.

### 4.3 Clinical implications and future directions

The findings underscore the critical need for early screening and intervention for hearing loss in children to mitigate the potential long-term cognitive impairment. The observed relationship between hearing loss duration and cognitive outcomes highlights the importance of timely management. Additionally, the protective effect of education suggests that targeted interventions and educational programs may help mitigate cognitive impairment in individuals with hearing loss. Consistently, a human study revealed that cognitively stimulating activities may enhance cognitive function in elderly individuals with hearing loss (Lin et al., 2023). Our animal studies also showed that environmental enrichment significantly improved cognitive function in the mice with hearing loss, highlighting its potential to enhance cognitive resilience.

While our human data showed no significant association between duration of childhood hearing loss and cognitive impairment (Table 1), the mouse experiments demonstrated progressive cognitive decline from P30 to P120 (Figure 3). This apparent discrepancy may reflect fundamental differences between species and experimental conditions. In humans, participants typically receive clinical interventions during their hearing loss period that may mitigate duration-dependent effects, whereas mice experienced complete auditory deprivation in a controlled environment without therapeutic intervention. Additionally, standardized cognitive tests may lack the sensitivity to detect subtle neuropsychological progression compared to behavioral paradigms used in mice. Furthermore, these methodological distinctions suggest that clinical rehabilitation strategies may modulate the longitudinal impact of hearing loss on cognition, potentially explaining the differential duration effects observed between clinical populations and controlled experimental models.

The present study had limitations, including a small human sample size and potential selection bias. Longitudinal studies are needed to establish causal relationships between hearing loss and cognitive impairment. Further research should also investigate the mechanisms by which hearing loss induced Tau phosphorylation, reduced synaptic protein concentrations, decreased neurogenesis, and increased neuroinflammation, and their interactions in the hearing loss-induced cognitive impairment. While this study demonstrates that environmental enrichment (EE) can effectively ameliorate hearing loss (HL)-induced cognitive deficits at the behavioral level, the underlying neurobiological mechanisms remain to be elucidated. Specifically, we did not investigate whether this behavioral improvement was associated with changes in synaptic plasticity markers, neuroinflammatory responses, or neurogenic activity in the hippocampus which leaded to important limitations of the current work. In future studies, we are systematically examining these potential mechanisms using a combination of molecular, histological and functional approaches to fully characterize the neuroprotective effects of EE.

## 5 Conclusion

This study highlights the significant impact of early-life hearing loss on cognitive outcomes in both patients and animal models. Findings from clinical and preclinical studies emphasize the importance of targeted interventions for hearing loss to enhance cognitive function. Future studies should further elucidate the cellular and molecular pathways involved and assess the therapeutic potential of neuroplasticity-enhancing strategies.

## Data Availability

The original contributions presented in the study are included in the article/supplementary material, further inquiries can be directed to the corresponding authors.
