# Supplementary material for: Early-life hearing loss induces persistent cognitive deficits: evidence from human data and a mouse model with environmental intervention
Source: Front Aging Neurosci. 2025 Sep 25;17:1662732. doi: 10.3389/fnagi.2025.1662732 (PMC12507710; doi:10.3389/fnagi.2025.1662732)

Early-life hearing loss induces persistent cognitive deficits: Evidence from human data and a mouse model with environmental intervention

Xuehua Zhou, MD , Huiqian Yu, MD, PhD , YiruWang, MD, Kaizheng Chen, MD , Xia Shen, MD, PhD Zhongcong Xie, MD, PhD

Western blot analyses for IL-1β and TNF-α

This Supplementary File presents the repeated Western blot analyses for IL-1β (Fig.S2.1 and Fig.S2.3) and TNF-α (Fig.S2.2 and Fig.S2.4))with molecular weight markers (PageRuler™ Prestained Protein Ladder, Thermo Scientific, #26616 or 26619) clearly indicated. These experiments were conducted under optimized conditions to address concerns regarding background interference and band clarity raised during the review process. The new results demonstrate improved band resolution and significantly reduced background, providing enhanced support for the quantitative expression data presented in the main text figures.

Figure Legends

Fig. S2. **Western blot images for IL-1β and TNF-α in hippocampal tissue.** These images are from repeated experiments with slightly improved clarity and reduced background compared to the original images in Figure 2D and E (Manuscript). Due to limited sample availability and antibody specificity, bands remain not fully optimal. The trends observed in these supplementary images are consistent with the quantitative data presented in the main figures.

Fig. S2.1. P30-IL-1β

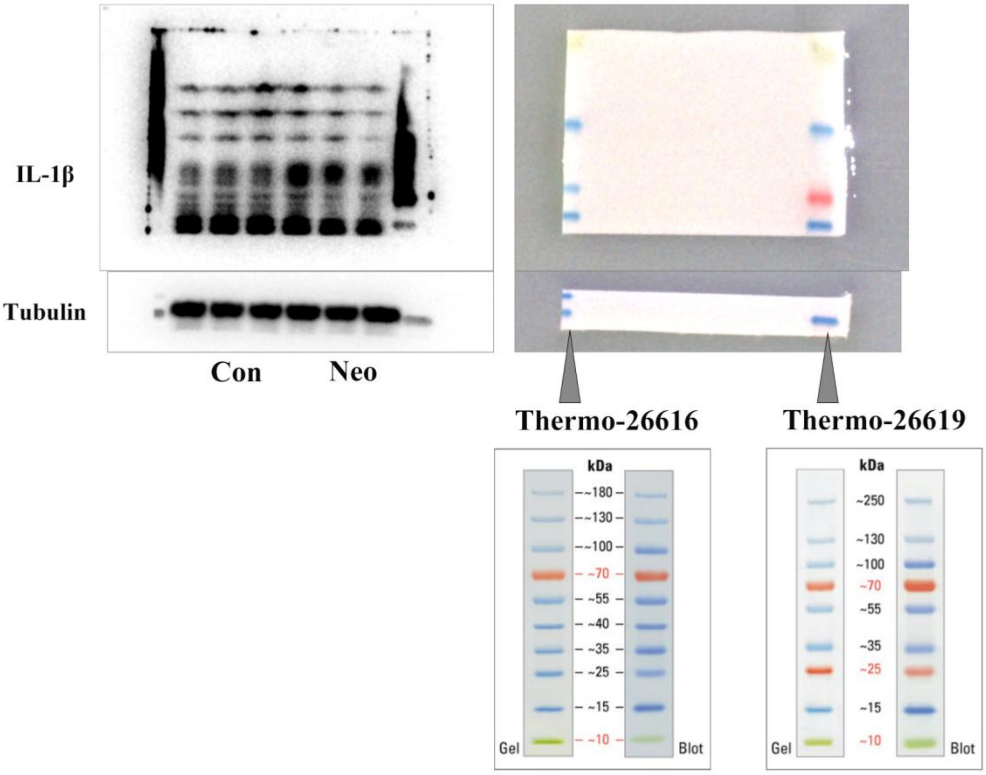

Fig. S2.2. P30-TNF-α

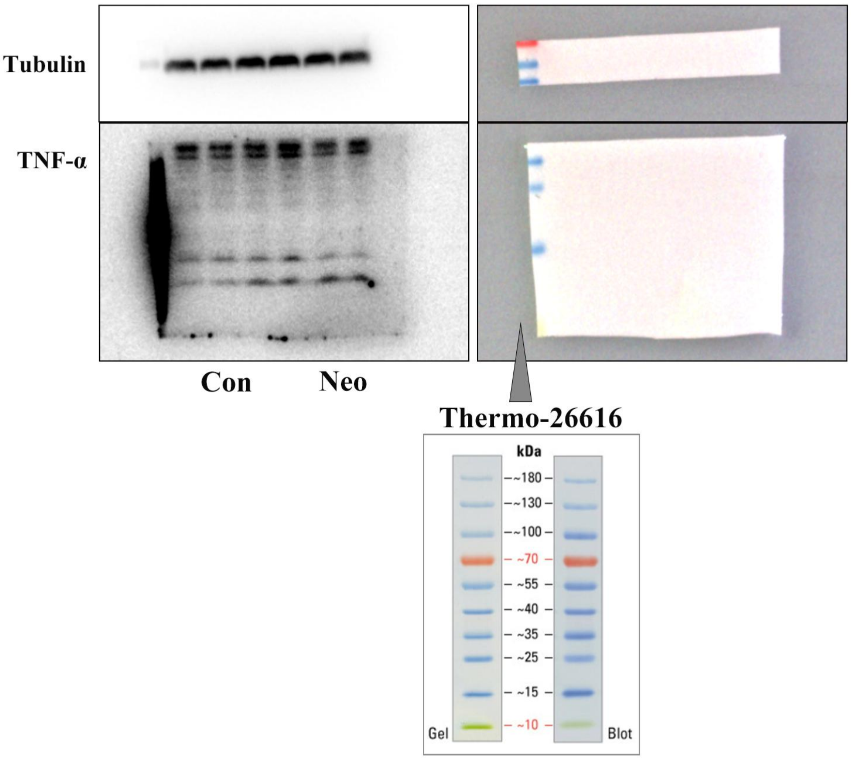

Fig. S2.3. P120-IL-1β

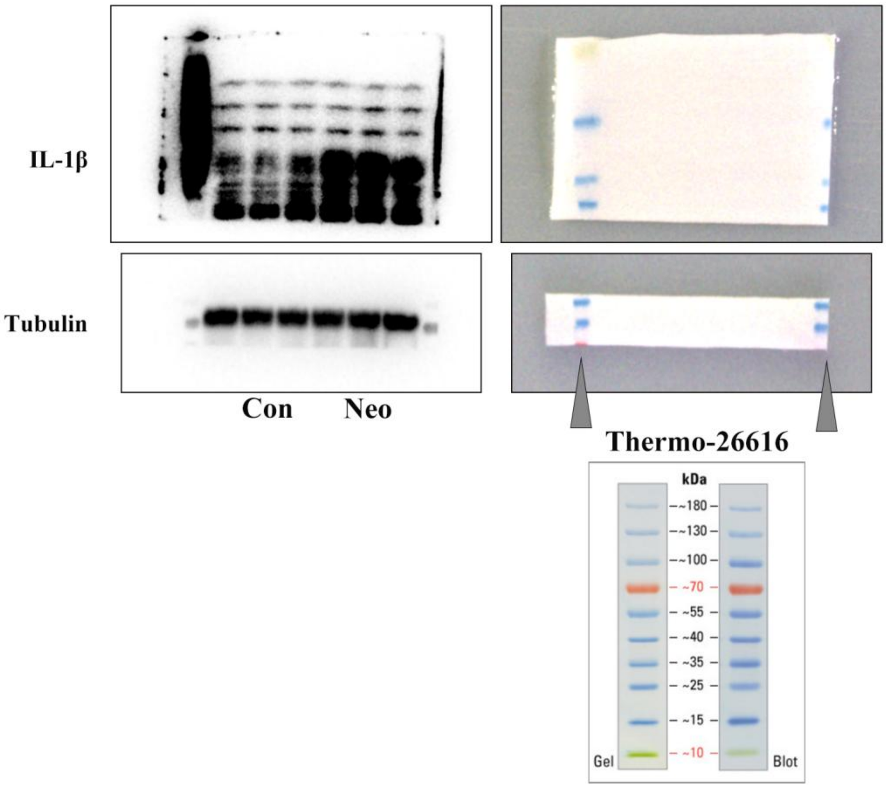

Fig. S2.4. P120-TNF-α

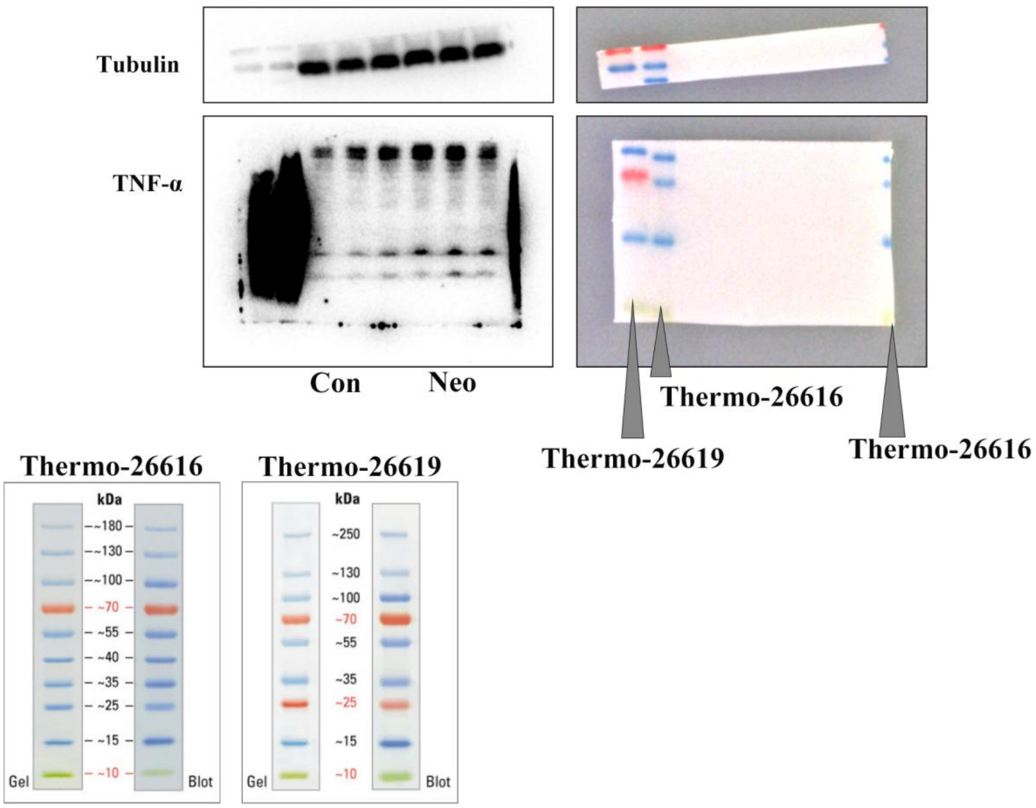

Supplement: Supplementary file 1 [file Data_Sheet_1.PDF]
