# Supplementary material for: Early-life hearing loss induces persistent cognitive deficits: evidence from human data and a mouse model with environmental intervention
Source: Front Aging Neurosci. 2025 Sep 25;17:1662732. doi: 10.3389/fnagi.2025.1662732 (PMC12507710; doi:10.3389/fnagi.2025.1662732)

Early-life hearing loss induces persistent cognitive deficits: Evidence from human data and a mouse model with environmental intervention

Xuehua Zhou, MD , Huiqian Yu, MD, PhD , , YiruWang, MD, Kaizheng Chen, MD , Xia Shen, MD, PhD, Zhongcong Xie, MD, PhD

Enriched environment (EE) Detailed Protocol

Mice were exposed to EE for 2 hours daily (10:00-12:00 AM) from P12 to P120 in 70×70×46 cm cages containing 5-6 items: plastic shelters , silent running wheels, PVC tunnels , wooden ladders , and puzzle feeders. All items were rotated 2-3 times weekly with 30% replacement to maintain novelty. Each item was sterilized regularly according to its material type via 70% ethanol wipes, UV germicidal irradiation or autoclave sterilization. HL+EE mice were group-housed (5/cage) to promote social interaction, while HL controls were housed 3/cage. Food included standard chow plus seeds hidden in puzzles. The photographic documentation of key enrichment items including climbing ladders, tunnels, shelters, running wheels, and wooden houses. In Supplementary Figure 1 (**Fig. S1**), we present several representative arrangements of enrichment items used in the environmental enrichment paradigm."

Fig. S1. Representative images of items used in the enriched environment.

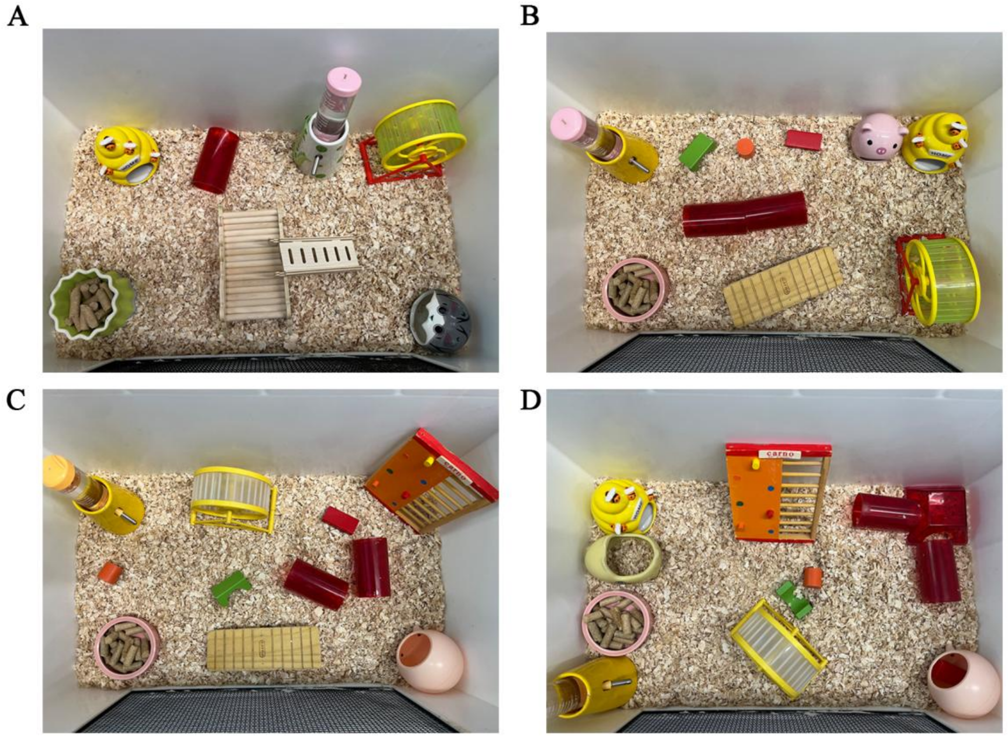

Supplement: Supplementary file 2 [file Data_Sheet_2.PDF]
