# Supplementary figures and images for: Early-life hearing loss induces persistent cognitive deficits: evidence from human data and a mouse model with environmental intervention
Source: Front Aging Neurosci. 2025 Sep 25;17:1662732. doi: 10.3389/fnagi.2025.1662732 (PMC12507710; doi:10.3389/fnagi.2025.1662732)

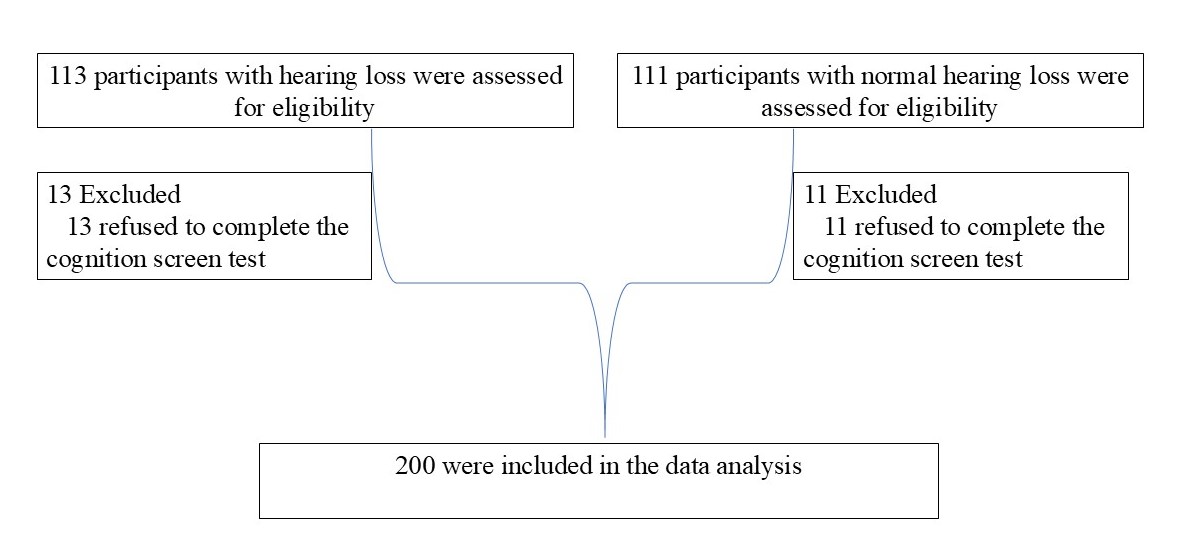

Supplement: Supplementary file 3 [file Image_1.JPEG]

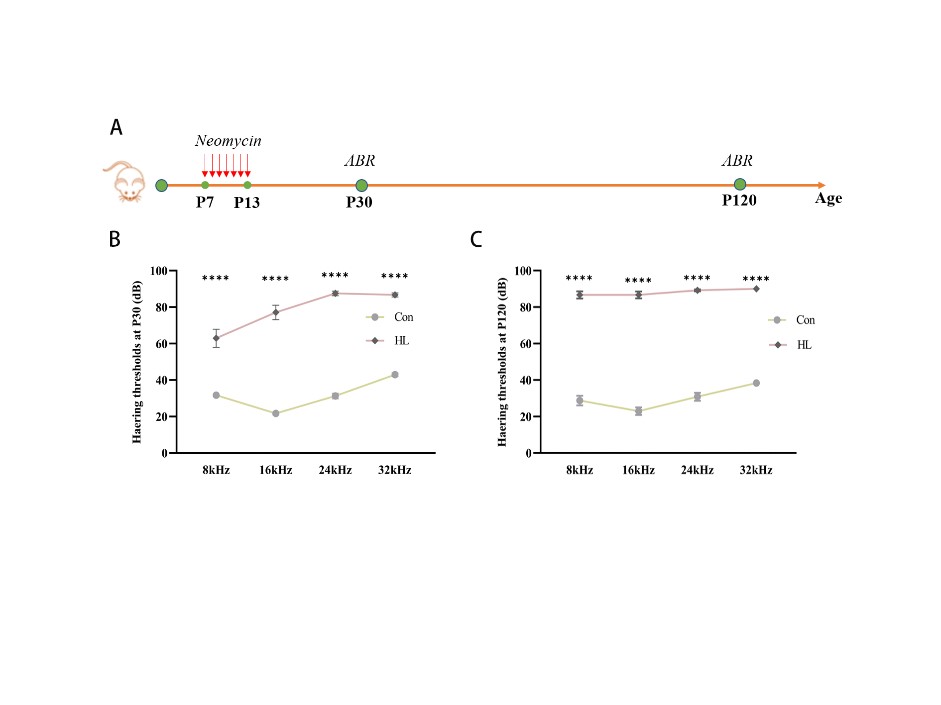

Supplement: Supplementary file 4 [file Image_2.JPEG]
